# A Review on N-Doped Carbon-Based Materials for the NH_3_-SCR Reaction

**DOI:** 10.3390/nano15201566

**Published:** 2025-10-15

**Authors:** Xueli Sun, Fangxiao Li, Yun Xu, Qian Zhang, Jingwen Ma

**Affiliations:** 1 State Key Laboratory of Low-Carbon Smart Coal-Fired Power Generation and Ultra-Clean Emission, China Energy Science and Technology Research Institute Co., Ltd., Nanjing 210023, China12059091@ceic.com (Y.X.); 12004291@ceic.com (Q.Z.); 2 School of Chemical and Environmental Engineering, China University of Mining and Technology (Beijing), Beijing 100083, China; 2210390103@student.cumtb.edu.cn

**Keywords:** nitrogen-doped carbon, NH_3_-SCR, NO*_x_* reduction, low-temperature activity

## Abstract

Nitrogen oxides (NO*_x_*), one of the major air pollutants, not only are key substances in forming acid rain and photochemical smog, but can also enter the stratosphere and damage the ozone layer to some extent. The selective catalytic reduction (NH_3_-SCR) technology has been widely utilized in industrial flue gas treatment for its efficient removal of NO*_x_*. In recent years, nitrogen-doped carbon materials (NC) have emerged as a novel type of environmentally friendly catalyst, showing outstanding performance in the low-temperature NH_3_-SCR reaction. This paper reviews the application advancements of nitrogen-doped carbon materials in the NH_3_-SCR reaction, with a focus on the catalytic mechanisms, modification strategies, and stability issues. This paper analyzes multiple improvement ideas, such as regulating metal types and distributions to achieve synergy effects, optimizing carrier loading, and designing morphology structures, and discusses how these measures jointly act to enhance the overall performance of the catalyst. Finally, solutions to the deactivation problem of NC catalysts are proposed, and the future research directions are explored to meet the increasingly stringent environmental protection requirements and promote the development of related technologies.

## 1. Introduction

Nitrogen oxides (NO*_x_*) are one of the major components of air pollutants, primarily originating from the emissions of fixed sources (such as power plants and factories) and mobile sources (such as automobiles) [1,2]. NO*_x_* not only gives rise to environmental issues like acid rain and photochemical smog but also undermines the ozone layer and poses serious threats to human health [1,2,3]. The commonly used flue gas denitrification technologies include SCR, selective non-catalytic reduction, oxidation–absorption denitrification, and the addition of denitrification additives [4,5,6,7]. Among these, SCR is the most widely applied and effective NO*_x_* reduction technology due to its technological maturity, operational stability, and high denitrification efficiency.

Currently, commercial SCR technology primarily employs NH_3_ as a reducing agent in a fixed-bed reactor, where NO*_x_* in the flue gas is catalytically reduced to harmless N_2_ under the action of a catalyst within a definite temperature window [8,9,10]. Selective catalytic reduction of NO*_x_* with NH_3_ (NH_3_-SCR) is currently the most widely applied approach for NO*_x_* elimination, prized for its high denitrification efficiency, superior N_2_ selectivity, large flue gas handling capacity, and controllable reaction conditions. Nevertheless, the reliance on ammonia as a reductant presents inherent challenges, including the risk of ammonia slip, the energy intensity of ammonia production, and the competition with its use as a fertilizer. These challenges drive the ongoing search for advanced SCR catalysts. Hence, the development of efficient SCR catalysts aims to address these limitations by enhancing low-temperature activity and selectivity, thereby minimizing ammonia consumption and improving the overall sustainability of the SCR process.

In recent years, nitrogen-doped carbon catalysts have attracted extensive attention from researchers as a novel type of environmental catalytic material. NC-based materials pertain to novel materials generated by connecting or integrating nitrogen atoms into the carbon material framework via chemical bonds, such as doped graphene [11,12], porous carbon [13,14,15], and carbon nanofibers [16,17]. Research indicates that numerous carbon-based catalysts can attain NO conversion rates of above 95% at low temperatures (150–250 °C), and certain catalysts have N_2_ selectivity exceeding 95% below 200 °C [18]. Furthermore, NC catalysts exhibit outstanding anti-poisoning performance, being capable of resisting the poisoning effects of impurities such as SO_2_, H_2_O, alkali metals, heavy metals, and fly ash in flue gas, thereby prolonging the service life of the catalysts. Simultaneously, as a metal-free catalyst, NC avoids the utilization of precious or rare metals and maintains relatively good performance under diverse flue gas conditions [19,20]. Utilizing NC as NH_3_-SCR catalysts can significantly reduce production costs, while not causing secondary pollution to the environment, conforming to the requirements of green development and possessing broad application prospects in future NO*_x_* emission reduction technologies. It is noteworthy that a primary advantage and research focus of NC catalysts lies in their promising performance within the low-to-moderate temperature window (typically 150–250 °C). This intrinsic characteristic positions them as a potential solution to overcome the limitations of conventional metal oxide catalysts, which often require higher operating temperatures and are consequently associated with greater energy consumption and operational costs. The subsequent sections of this review will, therefore, place particular emphasis on the strategies and mechanisms that enable efficient NH_3_-SCR activity on these carbon-based materials at reduced temperatures, directly addressing the pressing need for more energy-efficient and economically viable de-NO*_x_* technologies.

This review summarizes the recent progress in the development of NC-based catalysts for NH_3_-SCR. While several reviews have addressed carbon-based materials for various catalytic applications, there is a lack of systematic studies dedicated exclusively to NO*_x_* removal using NC catalysts. This paper focuses on the strategies developed to enhance the catalytic performance of NC materials, primarily through different types of nitrogen doping, morphology regulation, and their use as supports for other active materials. Additionally, this review discusses the effects of these strategies on improving the catalytic efficiency of NC catalysts in NH_3_-SCR. Finally, the paper presents future perspectives and potential research directions for further advancing NC-based catalysts in NO*_x_* abatement technologies.

## 2. Reaction Mechanism of NH_3_-SCR

The selective catalytic reduction reaction is a process in which nitrogen oxides (NO*_x_*) react with reductants (such as ammonia, urea, etc.) to generate nitrogen (N_2_) and water (H_2_O) via the action of a catalyst. The principal chemical reaction equation is presented as follows:

(1) $$4{NH}_{3}+4NO+O_{2}\to 4N_{2}+6H_{2}O$$

(2) $$4{NH}_{3}+2NO_{2}+O_{2}\to 3N_{2}+6H_{2}O$$

(3) $$4{NH}_{3}+2NO+{2NO}_{2}\to 4N_{2}+6H_{2}O$$

In the flue gas denitrification system, the disparity in the composition of nitrogen oxides will trigger diverse reaction pathways. When the molar ratio of NH_3_ to NO is 1:1, the reaction system adheres to the standard-SCR mechanism (Equation (1)), which necessitates the presence of oxygen. It is worth noting that when the molar ratio of NO to NO_2_ reaches 1:1, the fast-SCR reaction mechanism (Equation (3)) will be activated, and this reaction path possesses higher kinetic efficiency [21,22,23,24]. Consequently, the SCR reaction mechanism can be generalized as follows: (1) the indispensable role of O_2_ in low-temperature SCR; (2) the oxidation and adsorption of NO*_x_*; (3) the activation of NH_3_ on the catalyst. Different catalytic systems generate distinct intermediates due to the dissimilarities in active sites, thereby influencing the reaction pathways and SCR activity [25].

Adapa et al. [26] put forward two mechanism models for the catalytic oxidation of NO: namely, the Langmuir–Hinshelwood model (L-H model) and the Eley–Rideal model (E-R model).

The L-H model describes the synergistic reaction mechanism of NH_3_ and NO*_x_* on the catalyst surface. The process begins with the adsorption of gaseous NO (g) and O_2_ onto vacant active sites (Equation (4)). The adsorbed NO (ad) is subsequently oxidized by surface oxygen species (O*-NC), forming adsorbed NO_2_ (ad) or combining with surface oxygen to generate an intermediate complex such as ONO*-NC (Equations (5) and (6)). The primary role of NH_3_ is to act as a reducing agent, participating in the reaction via surface activation. Gaseous NH_3_ (g) adsorbs onto acidic sites (e.g., surface hydroxyl groups HO*-NC) on the catalyst and undergoes protonation to form the key ammonium ion species (NH_4_^+^-O*-NC) (Equation (7)). This NH_4_^+^ species serves as the primary active intermediate. It rapidly reacts with adsorbed NO_2_ (ad) to form an ammonium nitrite (NH_4_NO_2_) intermediate (Equation (8)), which is highly unstable and decomposes spontaneously into N_2_ and H_2_O, thereby achieving efficient nitrogen removal. Furthermore, the NH_4_^+^ species can undergo dehydrogenation on adjacent oxidizing sites (O*-NC) to generate a ·NH_2_ radical (Equation (9)). The derivation of this step is based on the oxidative activation of NH_4_^+^, where the surface oxygen abstracts hydrogen atoms, leading to the formation of the highly reactive ·NH_2_ radical. This NH_2_ radical can then attack the N atom in the ONO*-NC intermediate. The derivation of Equation (10) follows the mechanism of nucleophilic attack, where the nitrogen lone pair in ·NH_2_ attacks the nitroso nitrogen in ONO*, resulting in the formation of a N-N bond. The subsequent rearrangement and cleavage of the unstable diazenium diolate-like intermediate readily release N_2_ and H_2_O while regenerating the active site (O*-NC).

Although multiple pathways exist, the core route involves the formation of NH_4_^+^ from NH_3_, its reaction with NO_2_ via a “surface salt bridge” to form NH_4_NO_2_, and the subsequent decomposition to N_2_. The pathway via ·NH_2_ radical formation and its attack on ONO* species represents a parallel and complementary route [27].

The reaction process is as follows:

(4) $$NO_{\left(g\right)}\to {NO}_{\left(ad\right)}$$

(5) $${NO}_{\left(ad\right)}+O^{*}\text{-}NC\to {NO}_{2(ad)}+NC$$

(6) $${NO}_{\left(ad\right)}+O^{*}\text{-}NC\to {ONO}^{*}\text{-}NC$$

(7) $${NH}_{3(ad)}+{HO}^{*}\text{-}NC\to {NH}_{4}^{+}\text{-}O^{*}\text{-}NC$$

(8) $${NO}_{2\left(ad\right)}+{NH}_{4}^{+}\text{-}O^{*}\text{-}NC\to {NH}_{4}{NO}_{2}\to N_{2}+H_{2}O$$

(9) $${NH}_{4}^{+}+O^{*}\text{-}NC\to \cdot {NH}_{2}+NC+H_{2}O$$

(10) $${NH}_{2}+{ONO}^{*}\text{-}NC\to N_{2}+H_{2}O+O^{*}\text{-}NC$$

The E-R model holds that there exist differentiated interaction mechanisms between gaseous reactants and adsorbed species on the catalyst surface. Specifically, this mechanism encompasses three crucial stages: Firstly, NH_3_ is adsorbed onto the Lewis acid center to form a stable adsorbed NH_3_* (Equation (11)) [28,29]. Subsequently, gaseous NO molecules directly undergo an interfacial reaction with the surface NH_3_* without going through the adsorption process, generating an unstable intermediate complex (Equation (12)) [30]. Ultimately, this intermediate decomposes into gaseous N_2_ and H_2_O (Equation (13)), while simultaneously restoring the active sites of the catalyst (Equation (14)). The reaction process can be expressed as

(11) $$NH_{3(g)}\to {NH}_{3(ad)}$$

(12) $${NH}_{3(ad)}+O^{*}\text{-}NC\to \cdot {NH}_{2}+{OH}^{*}\text{-}NC$$

(13) $$\cdot {NH}_{2}+{NO}_{\left(g\right)}\to N_{2}+H_{2}O$$

(14) $$2{HO}^{*}\text{-}NC\to H_{2}O+O^{*}+2NC$$

O* represents reactive oxygen species.

**Figure 1:**
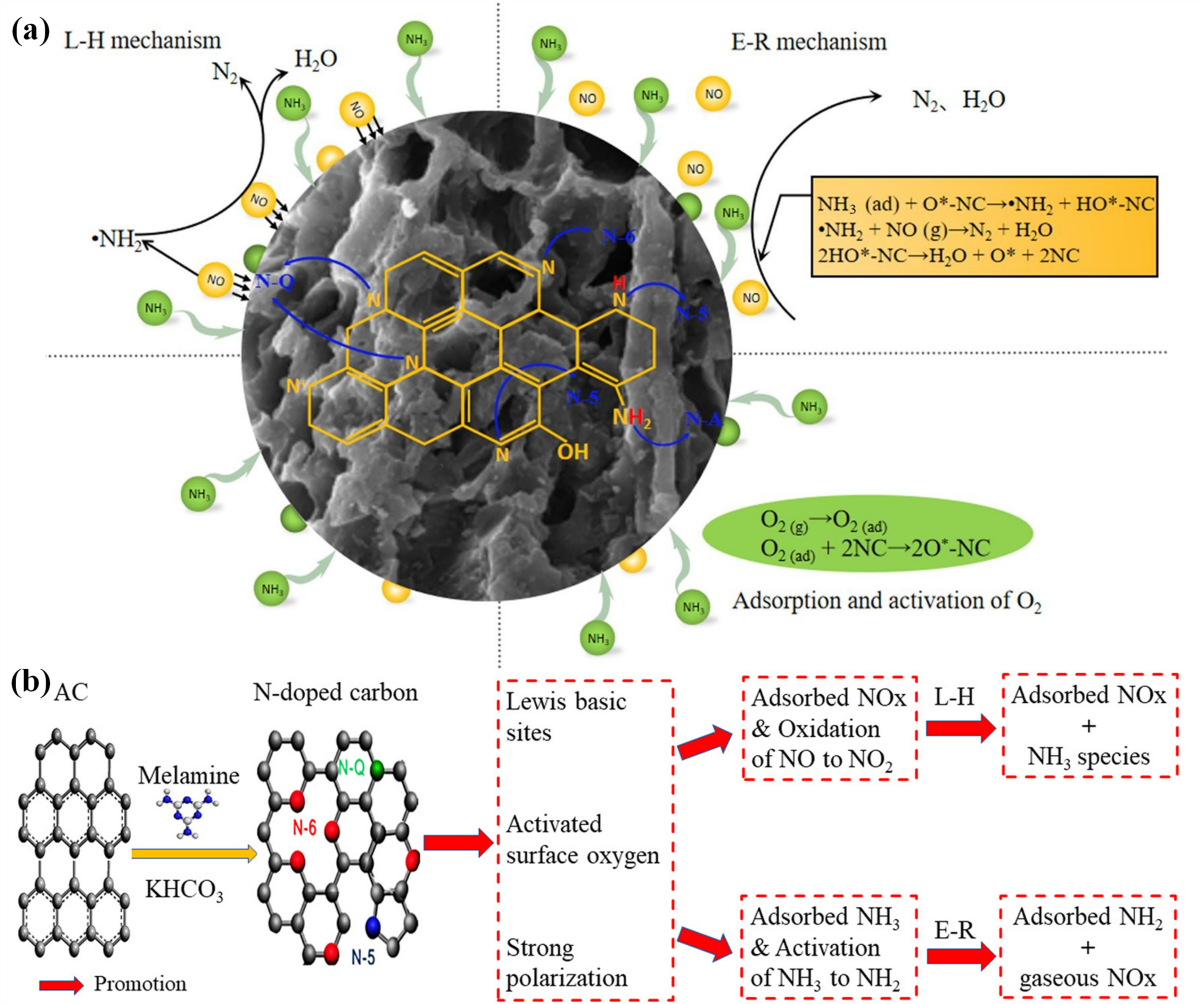
The L-H mechanism and E-R mechanism occurring on NC materials [32,34]. (**a**) Analysis of NH_3_-SCR path on N-doped biochar [32]. (**b**) The promotional NH_3_-SCR mechanism over N-doped carbons in [34].

Owing to the relatively low activation energy, the L-H mechanism is more prone to proceed at low temperatures [31]. Liu et al. [32] prepared a series of nitrogen-doped modified biochar using the cotton stalk as the raw material and examined the effect of different nitrogen-containing groups on the NH_3_-SCR. The results demonstrated that within the temperature range of 150–225 °C, the concentration of NO_2_ decreased rapidly with the increase in temperature, reaching the minimum of 0 ppm at 225 °C. This implies that as the temperature ascends, the denitrification pathway of biochar transforms from the “fast-SCR” reaction to the “standard-SCR” reaction. The steady state (approximately 0 ppm) was attained within 50 min after the removal of NO from the gas phase, indicating the existence of a dual-pathway synergy of L-H and E-R at the reaction interface. The former involves the surface redox of adsorbed NH_3_ and NO, while the latter originates from the direct reaction of gaseous NO and chemically adsorbed NH_3_*. In summary, both the E-R mechanism and the L-H mechanism are prevalently present in the low-temperature NH_3_-SCR of carbon-based catalysts [33]. Due to the complexity of flue gas circumstances and the diversity of the morphology and structure of NC catalysts, the mechanisms are different for different nitrogen-doped carbon catalysts (Figure 1) [32,34]. Consequently, the specific reaction mechanism of nitrogen-doped carbon catalysts in catalytic SCR still requires further in-depth investigation.

## 3. The Functional Mechanism of NC

The functional mechanism of nitrogen-doped carbon catalysts in the SCR mainly encompasses the following several aspects: regulation of active sites, optimization of the electronic structure, and adsorption and activation of ammonia molecules [35,36,37]. The following will elaborate on how these factors jointly act on the NH_3_-SCR reaction.

The local structural defects introduced by nitrogen doping modify the electronic structure of carbon materials, giving rise to a local electric field on their surfaces. Zhao [38] employed pyridinic N species as the active site model and doped them onto the surfaces of carbon nanotubes (CNTs) and graphene. The pyridinic N sites form a potent local electric field, protruding like a tip from the curved surface of CNTs, enriching the protons surrounding the adsorbed molecules and creating electron-rich defect sites, significantly enhancing the chemical adsorption capacity for NH_3_. Li [39] discovered that the carbon atoms bonded with pyrrolic N species are the active sites for the reaction, and the reaction activity rises with the increase in pyrrolic N sites; furthermore, nitrogen functional groups (such as pyridinic N or pyrrolic N) induce local electronic states below the Fermi level at carbon sites. These functional groups can act as proton donors, augmenting the surface acidity of carbon materials and concurrently reducing the activation energy barrier of NO.

Nitrogen doping can effectively modulate the surface characteristics of the catalyst. Li et al. discovered that the samples doped with N can effectively promote the conversion of NO to NO_2_ under low-temperature conditions and accelerate the dehydrogenation of NH_3_ molecules to generate highly active NH_2_* intermediates. This phenomenon can be ascribed to the electron interaction between the pyridinic N group and the adjacent carbon atoms, where the former, as a Lewis basic site, significantly enhances the adsorption capacity of NO*_x_*. Meanwhile, the introduction of N-containing groups also increases the number of oxygen vacancies on the catalyst surface. This occurs because the incorporation of nitrogen atoms, which have different valence states and atomic sizes compared to carbon, disrupts the local charge balance of the carbon matrix and induces lattice strain by altering the local electron density and introducing lattice distortions. To stabilize this modified electronic environment, the system tends to generate more oxygen vacancies by releasing partial lattice oxygen, thereby serving as effective charge compensation sites. The oxygen vacancies and surface oxygen-containing functional groups (such as C=O, -COOH) induced can thereby serve as redox mediators. They not only facilitate the low-temperature conversion of NO to NO_2_ but also accelerate the dehydrogenation of NH_3_ molecules, generating highly reactive NH_2_* intermediate species. These defect sites offer more adsorption active sites for NO molecules [40,41], thereby further enhancing the overall performance of the catalyst.

Carbon materials possess diverse microstructures, encompassing crystalline, amorphous, porous, and composite structures [42]. The interaction among multiple microstructures renders it infeasible to directly identify the origin of activity through experiments independently [43]. Research indicates that composite nanofibers fabricated by combining graphitic carbon nitride (g-C_3_N_4_) with a polyacrylonitrile precursor (PAN) via electrospinning technology can attain a NO conversion efficiency of 47.6% at room temperature [44], nearly doubling that of the unmodified samples. This performance enhancement primarily stems from two structural advantages: Firstly, the nitrogen-rich groups on the fiber surface can act as Lewis base sites, selectively capturing NH_3_ molecules and forming stable NH_3_* adsorption states, providing requisite active intermediates for the catalytic reaction [43,45,46]. Secondly, the disparity in charge distribution between N and adjacent C atoms within the material leads to an augmentation in structural disorder. This characteristic not only enlarges the pore volume but also enhances the degree of graphitization, thereby furnishing more active sites for NO molecule adsorption and facilitating the catalytic reaction process between NH_3_ and NO*_x_* [47]. Qu [48] doped various nitrogen-containing functional group substances (pyridinic N, pyrrolic N, quaternary N, etc.) into multi-level N-doped hierarchical porous activated coke (NHAC) for NO catalytic oxidation. The amorphous structure of NHAC makes it nearly impossible for NO molecules to be captured through pure physical adsorption. The introduction of nitrogen-containing functional groups can enhance the chemical adsorption capacity on the surface of the active coke, significantly elevating the NO adsorption capacity and facilitating the adsorption and stabilization of NO*_x_* molecules on the catalyst surface at low temperatures. Hence, accurately disclosing the influence of nitrogen functional groups on the NO*_x_* oxidation reaction necessitates a concerted consideration of the structural properties of carbon-based materials.

## 4. Nitrogen-Doped Carbon as Metal-Free Catalysts

Nitrogen-doped carbon materials play a crucial role in catalytic reactions owing to their vast specific surface area, which is conducive to the adsorption of NO*_x_* and NH_3_ molecules. It has been verified that introducing nitrogen groups into the carbon structure can generate a synergistic effect, thereby enhancing the catalytic performance and optimizing the NO*_x_* reduction efficacy [49,50]. The following will present nitrogen-doped carbon materials with different nitrogen groups and concentrations, as well as NC catalysts with distinct morphological structures.

### 4.1. Regulating the Concentration and Types of Nitrogen Doping

**Figure 2:**
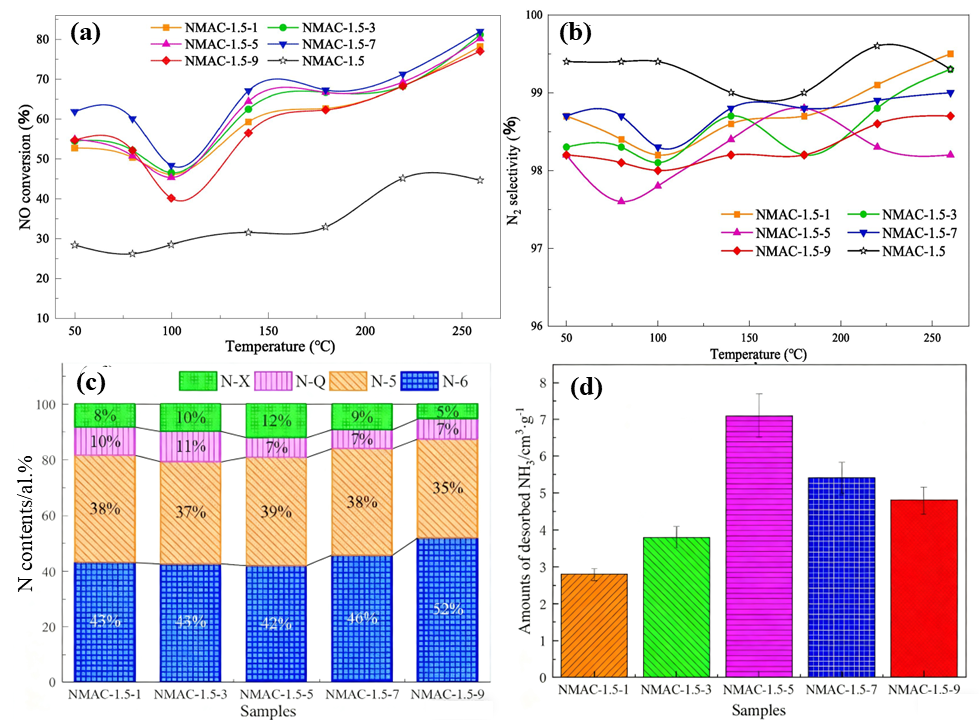
Low-temperature denitrification test of cotton stalk-based activated carbon with different nitrogen content ratios: (**a**) NO conversions; (**b**) N_2_ selectivity; (**c**) contents of different N groups over samples; (**d**) amounts of desorbed NH_3_ [32].

The nitrogen doping concentration exerts a crucial influence on the structural stability of the catalyst. Studies have indicated that an appropriate level of nitrogen doping can enhance the catalytic activity of the catalyst [51]. Nevertheless, an overly high nitrogen doping concentration might render the acidity on the catalyst surface overly intense, which would augment the adsorption capacity of the reactants [16], thereby resulting in difficulty in reactant desorption and consequently reducing the catalytic efficiency. Simultaneously, an excessive amount of nitrogen groups may have an impact on the stability of the catalyst surface structure [52,53]. These unstable nitrogen sites are prone to oxidation or reduction during the reaction process, leading to the deactivation of the catalyst. Liu [32] employed cotton straw as a precursor and modified it via urea impregnation to systematically investigate the influence of varying nitrogen doping ratios on the low-temperature denitrification performance of the resulting biochar. The sample label NMAC-1.5-y denotes nitrogen-doped modified biochar derived from MAC-1.5, where “1.5” refers to the H_3_PO_4_ impregnation ratio used in precursor preparation and “y” represents the urea-to-biochar mass ratio during N doping. The nitrogen-modified cotton straw biochar (NMAC) exhibited optimal low-temperature catalytic activity, achieving a NO conversion efficiency of 82% at 260 °C when the nitrogen doping ratio reached 7 (Figure 2). In the corresponding XPS analysis, N-6, N-5, N-Q, and N-X refer to pyridinic N, pyrrolic N, graphitic N, and pyridinic N-oxide, respectively.

**Figure 3:**
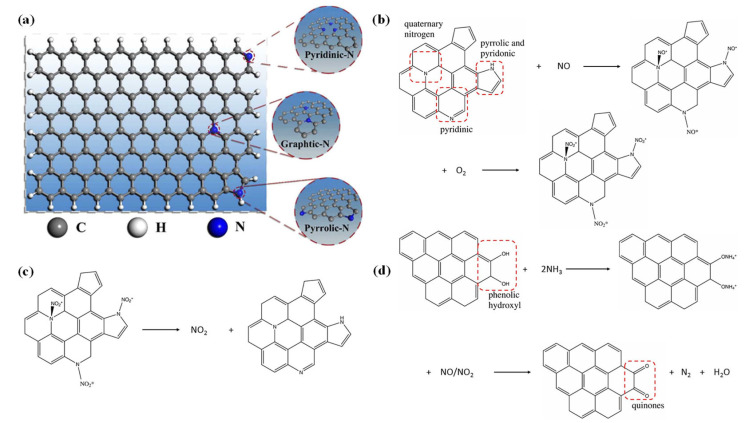
(**a**) Schematic illustration of the structure of different nitrogen species in nitrogen-doped carbon materials [55]; (**b**) conversion process of NO on pyridine, pyrrole, and quaternary nitrogen groups [56]; (**c**) desorption process of NO_2_ on AC [56]; (**d**) adsorption of NH_3_ on phenol hydroxyl groups and SCR reaction process [57].

Furthermore, the type of nitrogen doping is also a significant factor influencing the stability of the catalyst [54], which mainly includes pyridinic N, pyrrolic N, and graphitic N. As shown in Figure 3a [55], a schematic illustration of the structure of different nitrogen species (pyridinic N, pyrrolic N, and graphitic N) in nitrogen-doped carbon materials is presented. Figure 3b–d, respectively, illustrate the conversion process of NO on pyridine, pyrrole, and quaternary nitrogen groups, the desorption process of NO_2_ on carbon materials [56], and the adsorption process of NH_3_ and the SCR reaction process [57]. For example, Qu [48] doped various nitrogen-containing functional group substances (pyridinic N, pyrrolic N, graphitic N, etc.) into multi-level NHAC. Compared with the original carbon materials and pyridinic N- and pyrrolic N-doped carbon, the graphitic N-doped surface exhibits much stronger reactivity in the chemical adsorption of NO or O_2_, which attests that graphitic N plays a dominant role in enhancing the catalytic oxidation of NO. This is because the quaternary N significantly enhances the electron donor capacity of the carbon surface. Li et al. [58] systematically probed into the preparation and catalytic properties of nitrogen-doped porous carbon materials. They discovered that the RH/KOH/melamine (RH/M/K) material achieved a conversion efficiency of over 90% for the NH_3_-SCR reaction at 200 °C, demonstrating outstanding catalytic performance. Additionally, the activated carbon and melamine treated for 5 h (ACM-5) material prepared by Li [34] attained a NO*_x_* conversion rate of 52% at 150 °C, and its SCR activity was significantly superior to that of conventional activated carbon. The above studies indicate that nitrogen-containing groups play a vital role in catalytic reactions, providing a theoretical basis for the development of efficient SCR catalysts.

Different nitriding treatment approaches can also exert an influence on the catalytic performance of materials. The team of Sousa [57] successfully fabricated nitrogen-containing polymer precursor carbon xerogels through sol–gel technology, using urea and melamine as nitrogen sources. The experiments revealed that the introduction of nitrogen could considerably enhance the material’s catalytic efficacy. Meanwhile, the chemical properties and structure of the carbon xerogels were modulated by factors such as the pH value of the solution, the ratio of precursors, and the pyrolysis temperature. Under ambient temperature conditions, the NO removal efficiency of this material could arrive at 88%. The team further investigated Norit commercial activated carbon (AC) [59], systematically comparing the impacts of various nitriding processes (including nitric acid oxidation, room-temperature urea treatment, melamine treatment, and high-temperature urea treatment) on the catalytic performance. The results indicated that the catalytic performance of AC samples modified through nitriding was significantly enhanced. Among them, the samples treated with nitric acid oxidation and melamine exhibited the most outstanding performance, with the NO conversion efficiency reaching 88% at 25 °C.

A clear understanding of the specific functions of nitrogen species is key to catalyst design. The collective evidence indicates that pyridinic N groups are primarily responsible for the adsorption and oxidative activation of NO [34,57,58], a critical step for both L-H and fast-SCR pathways. Conversely, pyrrolic N functionalities enhance the surface acidity necessary for NH_3_ adsorption and its subsequent activation into NH_4_^+^ (for the L-H pathway) or ·NH_2_ species (for the E-R pathway) [32,41]. Graphitic N, while less directly involved in reactions, provides essential electronic conductivity and structural integrity [54]. Therefore, the overall catalytic efficiency is governed by the balanced co-existence and synergy of these nitrogen species.

### 4.2. Optimizing the Pore Structure of NC

During the design and optimization process of catalysts, the structure and surface characteristics of materials play equally crucial roles. Nitrogen-doped porous carbon materials have demonstrated remarkable performance enhancement effects. Through the rational adjustment of the pore structure of the materials [60] and the distribution of nitrogen groups, the selectivity and reaction rate of catalytic reactions can be effectively enhanced. Next, the specific applications and effects of the structural optimization strategies will be, respectively, explored to showcase their significant roles in the SCR reaction.

**Figure 4:**
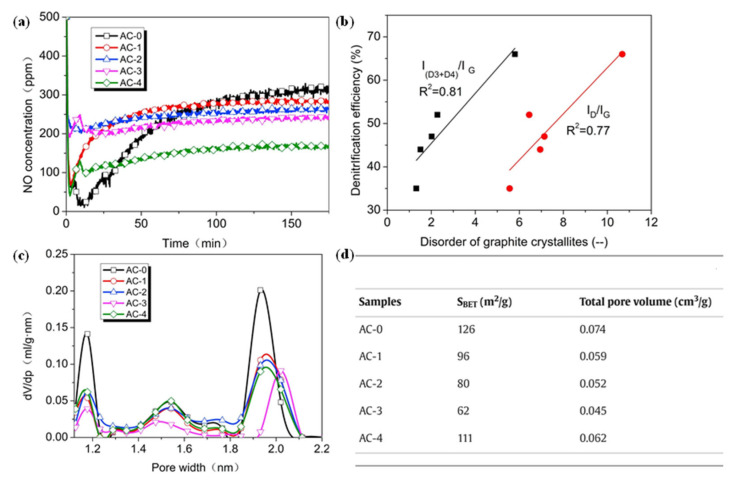
(**a**) Breakthrough curves for NO concentration on various AC samples; (**b**) effects of the disorder of graphite crystallites on denitrification efficiency; (**c**) pore size distribution on various AC samples; (**d**) specific surface area and total pore volume of AC samples [56].

The porous structure in nitrogen-doped porous carbon materials can not only stabilize the active intermediate species but also enhance the specific surface area of the carbon materials, thereby exposing more active sites [61], which is conducive to capturing nitrogen molecules, accelerating the synthesis rate of ammonia, and facilitating the SCR reaction. Lin et al. [56] modified activated carbon using four nitrogen-rich additives (pyrrole, pyridine, urea, and melamine) to prepare a series of samples labeled AC-0 (pristine), AC-1 (urea-modified), AC-2 (melamine-modified), AC-3 (pyrrole-modified), and AC-4 (pyridine-modified). The research discovered that the activated carbon treated with urea mainly generated graphitic N, while melamine mainly produced pyridinic N, pyrrole mainly produced pyrrolic N and graphitic N, and pyridine produced pyridinic N and graphitic N. By comparing the modification effects of these additives, it was found that their superiority decreased in the order of pyridine, pyrrole, melamine, and urea (Figure 4). Pyridinic N, pyrrolic N, and graphitic N can adsorb NO in the gaseous state, while phenolic hydroxyl groups serve as adsorption sites for NH_3_, thereby generating quinone and N_2_ and completing the SCR reaction. Hence, the existence of these nitrogen groups effectively promotes the denitrification efficiency of activated carbon. Further tests conducted by the team revealed that although the specific surface area and total pore volume of the modified activated carbon decreased, its denitrification efficiency was significantly enhanced. This was mainly attributed to the disorder of the graphite microcrystalline structure, which provided more active centers for the adsorption and reaction of NO and NH_3_. Moreover, the pore structure of the modified activated carbon varied with the type of additive, possibly due to the increase in the number of nitrogen functional groups in the micropore channels. These nitrogen-containing functional groups were capable of replacing carbon atoms in the graphite crystal structure with nitrogen atoms to form sp2-hybridized molecules and altering the main reaction pathways of the AC samples, combining with other atoms at the microcrystalline edges to form reactive centers, thereby significantly improving the denitrification efficiency.

## 5. Nitrogen-Doped Carbon Materials as Support for Metal Catalysts

Although nitrogen-doped carbon-based catalysts have demonstrated high efficiency in low-temperature catalytic reactions [32], challenges such as relatively poor stability, facile sintering at high temperatures, and low production yield remain. These issues can be addressed by forming composites with transition mentals or other additives and by optimizing morphological structure such as through coating strategies, thereby facilitating their industrial application. This section is dedicated to the strategy of using nitrogen-doped carbon materials as functional supports for transition metals, forming composite catalysts that exhibit superior low-temperature SCR activity.

### 5.1. The Synergistic Interaction Between Transition Metals and Nitrogen-Doped Carbon

Research indicates that when metal nanoparticles are adhered to carbonaceous materials, the composite materials typically demonstrate more excellent electrochemical performance. Simultaneously, nitrogen-doped carbon materials are capable of enhancing the dispersion of the metal active sites and forming a synergy with transition metals, fully exploiting the electron transfer capacity of transition metals, thereby conspicuously enhancing the activity and selectivity of the catalyst.

The structure and chemical properties of the support play a decisive role in the performance of the catalyst. Among them, nitrogen-doped carbon materials as catalyst supports can not only effectively disperse the metal active components and prevent their aggregation, which leads to reduced activity, but also adsorb acidic gases such as SO_2_ through their alkaline properties, thereby significantly enhancing the catalyst’s anti-sulfur-poisoning ability and overall catalytic performance. This modification strategy offers an effective idea for optimizing the performance of the catalyst. Zhao et al. [62] prepared a series of nitrogen-doped graphene (NG) and TiO_2_-supported MnO-CeO_2_ catalysts by the hydrothermal method. The results indicated that the introduction of NG significantly enhanced the NH_3_-SCR activity and SO_2_ tolerance of the catalyst at low temperatures. Specifically, the addition of NG decreased the crystallinity of MnO-CeO_2_, facilitating the uniform dispersion of MnO and CeO_2_ particles on the TiO_2_-NG support surface and their existence in multiple oxidation states. This structural characteristic further influenced the electronic state around the manganese species. Meanwhile, the nitrogen atoms in NG, as alkaline centers, not only adsorbed acidic gases such as NO, facilitating the electron transfer between NO molecules and the support surface, but also effectively adsorbed SO_2_ [49], thereby significantly enhancing the anti-poisoning ability of the Mn-based catalyst. Additionally, the introduction of NG provided more active sites for the catalyst, enhancing the adsorption capacity of reactants and further improving the catalytic activity. Li et al. [63] prepared an N-doped graphene/CoFe_2_O_4_ catalyst and preliminarily explored its intrinsic mechanism for promoting SCR activity. Tests revealed that within the temperature range of 200–300 °C, the NO*_x_* conversion rate of the N-doped graphene/CoFe_2_O_4_ catalyst could reach over 95%. The study demonstrated that nitrogen doping not only improved the dispersion of CoFe_2_O_4_, resulting in a more uniform distribution of the active components on the support, but also induced more acidic sites on the catalyst surface, optimizing the redox performance of the catalyst and significantly enhancing its denitration activity.

**Figure 5:**
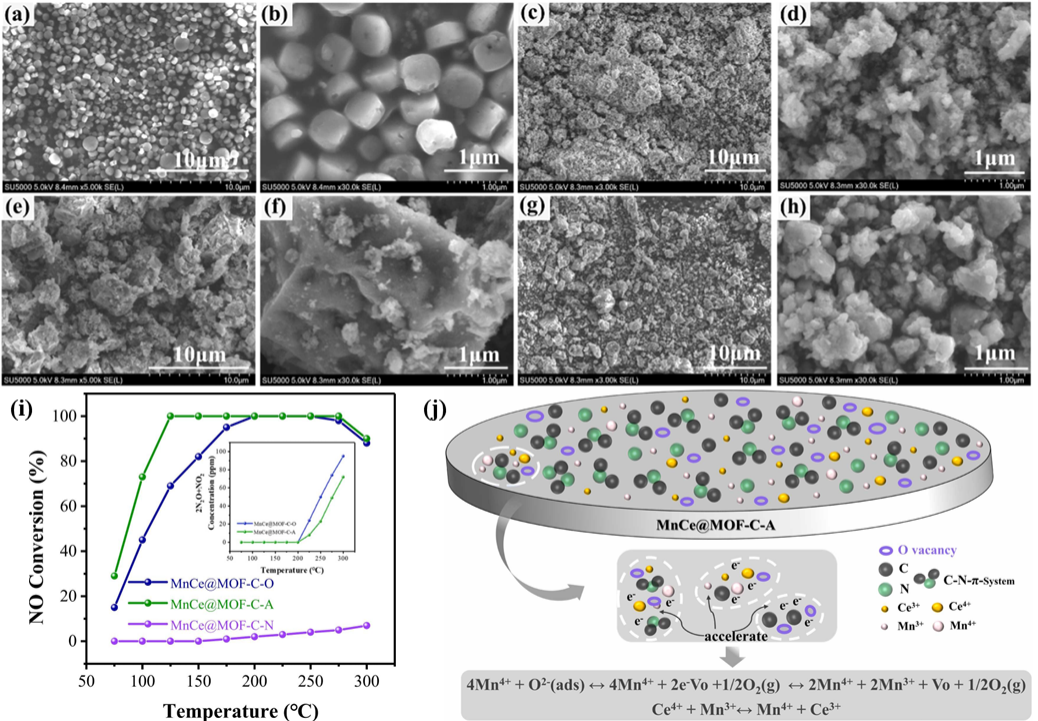
SEM images of MOF (**a**,**b**); SEM images of MnCe@MOF-C-O (**c**,**d**); (**e**,**f**) SEM images of MnCe@MOF-C-A; (**g**,**h**) SEM images of MnCe@MOF-C-N; (**i**) NO conversion rates of MnCe@MOF-C-O/A/N; (**j**) a schematic diagram of electron transfer on the surface of the catalyst MnCe@MOF-C-A [67].

By leveraging the outstanding electrochemical performance of NC composites, the electron transfer capacity of transition metals can be fully exerted [64,65]. Gao et al. [66] fabricated Ru catalysts supported on nitrogen-doped carbon nanotubes and discovered that the nitrogen-doped carbon nanotubes, owing to the electron-donating effect of surface nitrogen atoms and their abundant structural defects, strengthened the π bonds, thereby facilitating the uniform distribution of particles. In the Ru and carbon nanotube composites, the interaction between Ru particles and CNTs led to the electron transfer from Ru to CNTs, while the surface nitrogen atoms effectively compensated for this electron supply, increasing the electron density of Ru particles on the outer wall of CNTs. Compared with Ru particles loaded within the tube cavity, these outer-wall particles could adsorb and activate N_2_ molecules more effectively, further enhancing the catalytic efficiency of the catalyst. This finding is in line with the research results of Zhu [67], who successfully synthesized a series of nanocomposites (MnCe@MOF-C-O/A/N) by calcining MOF precursors in atmospheres with different oxygen concentrations (denoted as MnCe@MOF-C-O, MnCe@MOF-C-A, and MnCe@MOF-C-N, corresponding to calcination under oxygen, air, and nitrogen atmospheres, respectively). As shown in Figure 5, all samples experienced varying degrees of structural collapse and nanoparticle aggregation; when the damage was moderate, the active species became highly dispersed on the surface, which instead facilitated catalytic activity. Among them, MnCe@MOF-C-A (calcined in air) achieved 100% NO*_x_* conversion between 125 and 275 °C, primarily ascribed to its unique C–N structure in which nitrogen is bonded to two carbon atoms. This configuration promotes the formation of a p–π system, accelerates electron transfer, increases electrical conductivity, and thereby enhances catalytic activity. Moreover, the appropriate oxygen content not only enlarged the specific surface area of MnCe@MOF-C-A but also generated a greater number of oxygen vacancies on its surface.

Through the development of NC featuring special nanostructures and the optimization and modification of their structural defects, the synergistic effect between this material and metals can be effectively enhanced. Sung et al. [68] developed a nano-heterostructured support, incorporating Cu-Ce oxides into nitrogen-doped graphene quantum dots (CuCe-N-GQDs), and combined this substance with vanadate catalysts for the NH_3_-SCR reaction. When evaluated under simulated flue gas conditions containing SO_2_ (300 ppm) and H_2_O (10 vol%), the catalyst exhibited a NO*_x_* conversion efficiency as high as 91% and maintained stability for up to 52 h. The modification of N-GQD not only elevated the peak intensities of Brønsted acid sites and Lewis acid on the catalyst surface but also facilitated the formation of surface intermediate NO bridging species (nitrite and nitrate), thereby synergistically enhancing the NO*_x_* adsorption capacity of the catalyst surface. NO_2_ with a higher adsorption rate is more prone to react with adsorbed NH_3_ to form H_2_O and N_2_, thereby accelerating the SCR reaction process. Furthermore, CuCe-N-GQDs improved the coordination of NH_3_ and NH_4_^+^ ion species via an electronic effect, further strengthening the low-temperature SCR activity of the catalyst.

**Figure 6:**
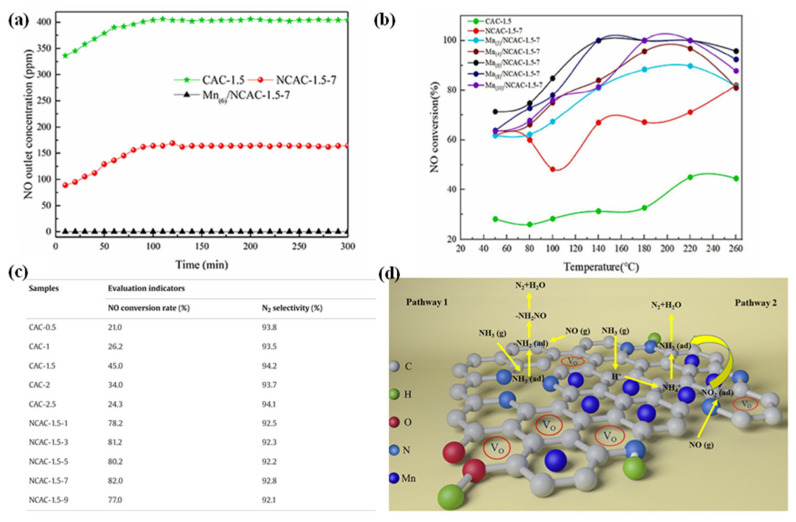
(**a**) NO concentration variation curves for different samples at the reaction temperature of 180 °C; (**b**) steady-state NO conversions at different temperatures; (**c**) NO conversion and N_2_ selectivity of cotton stalk biochar and nitrogen-doped cotton stalk carbon; (**d**) a schematic diagram of the reaction mechanism of Mn-modified nitriding cotton stalk biochar [72].

The introduction of nitrogen-containing groups enhances the catalytic performance by increasing the quantity and distribution of surface oxygen-containing functional groups, which then exert a synergistic effect with transition metals [69]. Feng [70] prepared a series of Mn-doped nitrogen-doped cotton stalk biochar catalysts, as shown in Figure 6. CAC-x denotes biochar with a H_3_PO_4_ impregnation ratio of x:1 (x = 0.5, 1, 1.5, 2). NCAC-x-y represents N-doped CAC-x with a urea-to-biochar mass ratio of y:1 (y = 1, 3, 5, 7, 9). Mn(z)/NCAC-x-y indicates Mn loading of z wt% (z = 2, 4, 6, 8, 10) on the specified support. The NO removal rate of the treated non-nitrogen-containing cotton straw biochar (CAC-1.5) was less than 45% throughout the entire temperature range; however, after nitrogen doping, the NO removal rate of NCAC-1.5 reached the maximum value (82%), and when NCAC-1.5 was further loaded with Mn, the NO removal rate of this sample approached 100% within the temperature range of 140–220 °C. The existence of nitrogen groups promotes the generation of oxygen vacancies and enhances the oxygen mobility of the catalyst, and the increased surface oxygen-containing functional groups synergistically interact with the main active component Mn_2_O_3_, further facilitating the oxidation of NO to NO_2_ and promoting the NH_3_-SCR reaction. Simultaneously, the introduction of nitrogen groups can effectively improve the thermal stability of carbon materials, addressing the potential thermal failure of transition metals under high-temperature conditions and thereby further enhancing their catalytic performance. Anna [71] incorporated Cu, Co, and Ag into nitrogen-containing activated carbon (AC), respectively. Among them, Co and Cu existed in the form of CoO and CuO microcrystals in the samples, while Ag existed in the form of large metal microcrystals. Through testing, it was found that the Co sample exhibited high catalytic activity at low temperatures, while the Cu sample had the best catalytic activity at high temperatures, with a NO conversion rate of approximately 80% at 140 °C, and the NO conversion rate could be increased to 100% when the temperature rose above 180 °C. Nevertheless, the thermal instability of carbonaceous materials at high temperatures is quite significant, and the presence of transition metals may accelerate the gasification of carbon, thereby leading to the thermal failure of the catalyst. Studies have shown that the deposition of nitrogen-containing functional groups not only improves the thermal stability of carbonaceous materials (the thermal stability of the materials can reach 260 °C), but also effectively inhibits the oxidation of carbon, acting as NO adsorption sites and synergistically exchanging electrons with transition metals [72], further enhancing the NO removal efficiency (92%).

### 5.2. Dual-Atom-Doped Carbon as the Support

Apart from metal doping, multi-element co-doping (such as S, P, etc.) is also widely employed for the optimization of catalyst performance. Co-doping can further regulate the electronic structure of the catalyst, augment the quantity of active sites, and ameliorate its morphological structure, thereby elevating the adsorption capacity and reaction activity of the catalyst.

**Figure 7:**
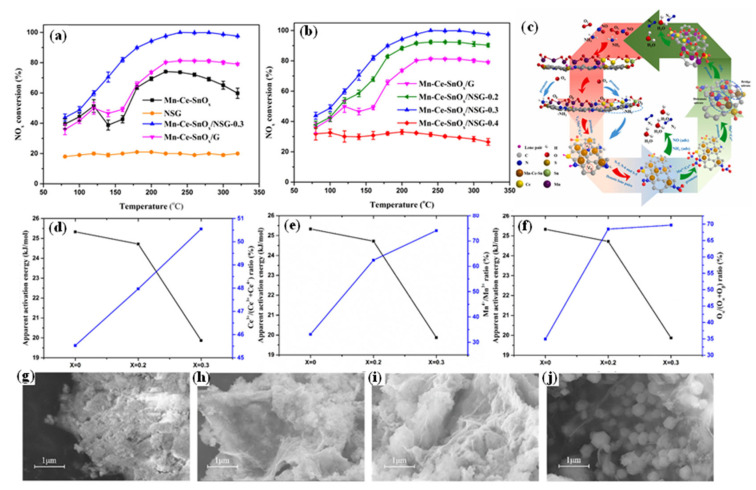
(**a**,**b**) NO*_x_* conversion; (**c**) promoting effect of N, S co-doped over Mn-Ce-SnO_x_/NSG-0.3 in L-H mechanism; (**d**–**f**) relationship between Ce^3+^/(Ce^3+^ + Ce^4+^), Mn^4+^/Mn^3+^, O_α_/(O_α_ +  O_β_), and Ea over Mn-Ce-SnO_x_/NSG-0.3; (**g**–**j**) FESEM images of Mn-Ce-SnO_x_/G and Mn-Ce-SnO_x_/NSG-X (X  =  0.2, 0.3, and 0.4) [73].

Zheng et al. [73] fabricated a series of N/S-co-doped graphene catalysts (denoted as Mn-Ce-SnO_x_/NSG-X, where X represents the mass content of thiourea used during synthesis, with values of 0.2, 0.3, and 0.4) through in situ synthesis to explore the influence of N/S co-doping on catalytic performance. As shown in Figure 7, the Mn-Ce-SnO_x_/NSG-0.3 catalyst presented outstanding SCR activity at low temperatures, with the conversion rate of NO*_x_* reaching 100% at 240 °C. The improvement in the activity of this catalyst was ascribed to the Lewis acid sites resulting from N/S co-doping, as well as the optimization of the ratios of Ce^3+^/(Ce^3+^ + Ce^4+^), Mn^4+^/Mn^3+^, and O_α_/(O_α_ + O_β_). These alterations effectively decreased the apparent activation energy and facilitated the adsorption and oxidation of NO. Furthermore, N/S co-doping also ameliorated the morphology and structure of the catalyst. It was discovered that after the addition of N/S, the surface transformed from a disordered stacking structure to a uniform and dense cotton-like structure, which was more favorable for the conversion of NO*_x_*.

The doping of multiple elements in NC composite materials enables not only a better exertion of the catalytic capabilities of each element but also a full utilization of the merits of NC. The synergy between the two makes their catalytic performance surpass that of using any single material independently. Despite the remarkable progress made in this aspect of research, improvements are still requisite in terms of the uniformity of nitrogen doping, stability, and large-scale preparation. Future studies will concentrate on precisely regulating the distribution and type of nitrogen doping to further enhance the comprehensive performance of the catalyst and promote its extensive application in fields such as environmental protection and energy.

### 5.3. Encapsulation Strategy

Coating the surface of metal nanoparticles with nitrogen-containing nanocarbon materials can form an onion-like structure of “metal core–surface carbon layer”. This strategy can not only effectively reduce the loss and leaching of active metals but also significantly enhance the stability of the catalyst.

**Figure 8:**
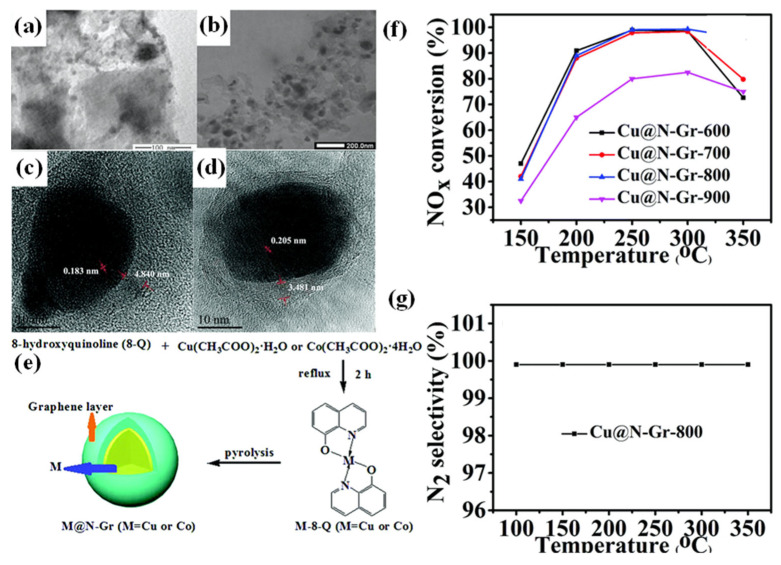
TEM images of Cu@N-Gr-800 (**a**) and Co@N-Gr-800 (**b**). HRTEM images of Cu@N-Gr-800 (**c**) and Co@N-Gr-800 (**d**). (**e**) A schematic illustration of the core–shell architecture where Cu and Co nanoparticles are encapsulated within N-doped graphene (Cu@N-Gr and Co@N-Gr). (**f**) NH_3_-SCR activity of Cu@N-Gr-600, Cu@N-Gr-700, Cu@N-Gr-800, and Cu@N-Gr-900. (**g**) N_2_ selectivity of Cu@N-Gr-800 [74].

As depicted in Figure 8, Li et al. [74] synthesized core–shell catalysts by coating Cu and Co nanoparticles with N-doped graphene (Cu@N-Gr and Co@N-Gr). The Cu@N-Gr-X (where X = 600, 700, 800, 900) denotes the calcination temperature applied during synthesis. The research results indicated that Cu@N-Gr-800 and Co@N-Gr-800 (Cu@N-Gr/Co@N-Gr calcined at 800 °C for 2 h) displayed higher NO*_x_* conversion rates. Particularly, Cu@N-Gr achieved a NO conversion rate of up to 89% within the temperature range of 200–350 °C. This was ascribed to the graphene shell effectively preventing the aggregation of Cu and Co nanoparticles, thereby highly dispersing the active sites on the surface. Furthermore, the Cu nanoparticles encapsulated in the graphene shell exhibited a lower reduction temperature than the unencapsulated Cu nanoparticles, suggesting that the synergy between graphene and Cu nanoparticles enhanced the reducibility of Cu@N-Gr-800 and its catalytic performance in the NH_3_-SCR reaction.

Through this encapsulation strategy, the performance of the catalyst was significantly improved, providing a more stable and efficient catalytic system for practical applications.

## 6. Discussion Regarding the Stability of NC

NCs possess remarkable potential as catalysts in the SCR reaction; however, they encounter deactivation issues during long-term usage. This is primarily manifested in the loss of catalyst active sites and the deterioration of catalytic performance. The influencing factors encompass nitrogen doping concentration, nitrogen group types, and operating temperature, among others [75]. Through in-depth investigations of these factors, researchers have put forward numerous optimization strategies to enhance the stability of the catalyst and retard deactivation.

The catalytic activity and stability of nitrogen-doped carbon materials are intimately associated with their surface acidity. The incorporation of nitrogen groups can augment the acidity of the catalyst, thereby enhancing its adsorption capability for NO and upgrading the catalytic performance [76]. Nevertheless, this effect might induce catalyst deactivation in the long run. In the SCR reaction atmosphere, there are frequently some corrosive substances, such as SO_2_ and H_2_O. These substances react with the nitrogen groups in the nitrogen-doped carbon materials to form acidic products (e.g., hydrogen bisulfate or hydrogen nitrate), which neutralize the nitrogen groups on the catalyst surface and accumulate on the catalyst surface, gradually occupying the active sites, thereby resulting in the deterioration of the catalyst’s activity.

To tackle this problem, researchers have put forward multiple strategies to enhance the stability of nitrogen-doped carbon materials, ensuring their favorable catalytic performance during prolonged reaction processes. Firstly, by optimizing the concentration of nitrogen doping and the types of nitrogen groups, the stability of the catalyst can be prominently improved [51,16,52,53]. An appropriate nitrogen doping concentration can maintain the catalytic activity while reducing the reaction probability with corrosive substances, and the choice of nitrogen group types is conducive to enhancing the corrosion resistance of nitrogen-doped carbon materials in adverse environments. Secondly, the introduction of metal elements or other auxiliary elements represents another effective approach to enhance the stability of the catalyst. The synergy between metal elements and nitrogen groups can strengthen the thermal stability of the catalyst, effectively inhibiting the damage inflicted by high temperatures and corrosive substances. These metals or auxiliary elements not only offer additional active sites but also, by forming metal–nitrogen–oxygen complexes, further enhance the catalyst’s tolerance to acidic products, thereby decelerating the deactivation process. Additionally, surface modification techniques, such as in situ reduction and surface oxidation, are also crucial means for enhancing the stability of the catalyst. Through these surface modification methods, the surface structure of the catalyst can be ameliorated, enhancing its resistance to corrosive substances and thereby elevating its anti-deactivation capacity. For example, in situ reduction can eliminate some oxides on the catalyst surface, thereby enhancing the reducibility of the catalyst, while surface oxidation contributes to enhancing the stability of the catalyst in oxidative atmospheres. These technical measures not only improve the surface structure of the catalyst but also effectively lower the deactivation rate of the catalyst during the reaction process.

## 7. Future Prospects and Challenges Ahead

The SCR technology, serving as a crucial means for mitigating nitrogen oxide (NO*_x_*) emissions, has been extensively utilized in transportation, industrial boilers, and power generation. Nevertheless, conventional SCR catalysts encounter numerous challenges. In recent years, nitrogen-doped carbon catalysts, armed with their remarkable catalytic performance and relatively lower cost, have gradually emerged as a research focus. Despite the fact that nitrogen-doped carbon catalysts have exhibited favorable catalytic performance in SCR reactions, there remain several issues that demand immediate solutions:

1. Carbon materials are prone to sintering at high temperatures, thus losing their catalytic activity. The nitrogen sites on carbon materials are especially susceptible to deactivation in high-temperature environments. Meanwhile, nitrogen-doped carbon materials are prone to deactivation and poisoning by SO_2_ and H_2_O after long-term SCR reactions. Future improvements are required in this aspect, such as optimizing the nitrogen doping mode or introducing appropriate carriers to enhance the thermal stability of the catalyst.
2. The homogeneity of nitrogen doping exerts a crucial influence on the performance of the catalyst. Nevertheless, the current nitrogen doping approaches still exhibit a certain degree of inhomogeneity. In the future, it is necessary to explore and develop more efficient nitrogen doping technologies to improve the homogeneity of the catalyst, with the aim of achieving a higher nitrogen doping amount and a more uniform nitrogen distribution, thereby enhancing the catalytic performance.
3. Despite the existence of numerous and outstanding modification approaches for nitrogen-doped carbon materials at present, the catalytic mechanism still requires in-depth investigation; in particular, the specific functions of different types of nitrogen sites in catalytic reactions remain ambiguous. Future studies need to profoundly explore the catalytic mechanism of nitrogen-doped carbon catalysts and deeply analyze the specific roles of different nitrogen sites in the SCR reaction.

## Data Availability

No new data were created or analyzed in this study.
